# Differences in breast cancer characteristics and outcomes between Caucasian and Chinese women in the US

**DOI:** 10.18632/oncotarget.3666

**Published:** 2015-03-26

**Authors:** Dan-Na Chen, Chuan-Gui Song, Qian-Wen Ouyang, Yi-Zhou Jiang, Fu-Gui Ye, Fang-Jing Ma, Rong-Cheng Luo, Zhi-Ming Shao

**Affiliations:** ^1^ Department of Breast Surgery, Affiliated Union Hospital, Fujian Medical University, Fuzhou, China; ^2^ Cancer Center, Traditional Chinese Medicine-Integrated Hospital, Southern Medical University, Guangzhou, China; ^3^ Department of Breast Surgery, The Third Hospital of Nanchang, Nanchang, China; ^4^ Department of Breast Surgery, Key Laboratory of Breast Cancer, Fudan University Shanghai Cancer Center, Shanghai Medical College, Fudan University, Shanghai, China

**Keywords:** breast cancer, clinicopathological characteristics, prognosis, Chinese, Caucasian

## Abstract

Chinese breast cancer patients living in the United States (US) can experience different disease patterns than Caucasians, which might allow for predicting the future epidemiology of breast cancer in China. We aimed to compare the clinicopathologic characteristics and outcomes of Caucasian and Chinese female breast cancer patients residing in the US. The study cohort consisted of 3868 Chinese and 208621 Caucasian women (diagnosed from 1990 to 2009) in the US Surveillance, Epidemiology, and End Results (SEER) database. Compared with the Caucasian patients, the US-residing Chinese patients had a younger age at diagnosis and a higher family income, remained married longer, and more frequently lived in metropolitan areas. Other tumor characteristics were similarly distributed between the two races. Compared with the Caucasians, the Chinese patients had a significantly improved overall survival (OS) but similar breast cancer-specific survival (BCSS). Our analysis suggested that US-residing Chinese patients had significant differences in age, family income, marital status and area of residence, compared with their Caucasian counterparts. No significant disparities were noted in BCSS between the two races, whereas the Chinese patients had a significantly better OS. These findings warrant further investigation and should be considered in the screening and treatment of breast cancer.

## INTRODUCTION

It has been well established that racial disparities exist in the incidence, mortality, and survival of patients with breast cancer. In China, breast cancer has a lower incidence and mortality compared with developed Western countries [1]. However, Chinese patients appear to have worse survival rates than Western women [2]. These discrepancies might be due to differences between the genetic backgrounds of Asians and Caucasians, as well as social-environmental factors, such as economic status, culture, lifestyle, and dietary habits. In addition, the incidence of breast cancer in China has been increasing rapidly [2, 3], and some researchers have speculated that the incidence curve will be similar to that in Western countries in the future [1], possibly due to an increase in economic status and alterations in lifestyles.

When Chinese people immigrate to high-income Western countries such as the United States (US), they frequently change their lifestyles to adapt to Western culture [4]. Once immigrants have left their native countries of origin, they might expose themselves to breast cancer risk factors similar to those of their Caucasian counterparts [5], indicating that data from Chinese populations cannot be extrapolated to Chinese immigrants living in the US. To our knowledge, there have been no studies specifically examining all of the available demographic, clinicopathologic, and outcome-related breast cancer parameters in the population-based Surveillance, Epidemiology, and End Results (SEER) database for the Chinese-American cohort. Because Chinese Americans are the largest Asian subgroup in the US and 76% are immigrants [6], the SEER database could be used to compare the breast cancer survival rates of Caucasians and Chinese Americans living in the US to determine potential factors affecting these two races.

## RESULTS

### Descriptive Statistics

With a median follow-up time of 74 months, a total of 3868 Chinese and 208621 Caucasian women were identified in our analysis, according to the inclusion and exclusion criteria stated in METHODS. Among these patients, 378 (9.77%) died from breast cancer and 531 (13.73%) died from all causes among the Chinese individuals, whereas 21641 (10.37%) patients died from breast cancer and 40698 (19.51%) died from all causes among the Caucasians. All of the demographic and tumor characteristics are shown in Table 1.

**Table 1:** Clinicopathologic characteristics of Caucasian and Chinese female breast cancer patients residing in the US from the SEER database

Characteristics	No. (%) of Patients		P^a^
	Chinese	Caucasian	
Total	3,868	208,621	
Age (mean) (years)	53.60	57.30	
Age at Diagnosis (years)			**<0.01**
20-39	407(10.52)	14,680 (7.04)	
40-59	2,305 (59.59)	103,119 (49.43)	
60-79	1,156 (29.89)	90,882 (43.56)	
Year of Diagnosis			**<0.01**
1990-1999	1,011 (26.14)	48,319 (23.16)	
2000-2009	2,857 (73.86)	160,302 (76.84)	
Marital Status			**<0.01**
Married	2,876 (74.35)	135,306 (64.86)	
Not married^b^	992 (25.65)	73,315 (35.14)	
Family Income (mean) ($)	83,231.58	73,049.72	
Family Income ($)			**<0.01**
<61,790	106 (2.74)	52,872 (25.34)	
61,790-69,700	962 (24.94)	52,132 (24.99)	
69,700-85,140	631 (16.31)	49,982 (23.96)	
≥85,140	2,169 (56.08)	52,638 (25.23)	
County Type			**<0.01**
Metropolitan	3,676 (95.04)	186,052 (89.18)	
Nonmetropolitan	192 (4.96)	22,571 (10.82)	
Laterality			0.75
Left	1,951 (50.44)	105,747 (50.69)	
Right	1,917 (49.56)	102,851 (49.30)	
Tumor Size (cm)			0.10
0-2	2,498 (64.58)	137,715 (66.01)	
2-5	1,161 (30.02)	59,339 (28.44)	
>5	209 (5.40)	11,567 (5.54)	
LN Status			0.09
Negative	2,523 (65.23)	139,399 (66.82)	
1-3 positive	896 (23.16)	46,814 (22.44)	
3 positive	446 (11.53)	22,314 (10.70)	
AJCC Stage			0.09
I	1,910 (49.37)	106,580 (51.08)	
II	1,439 (37 20)	75,584 (36.23)	
III	519 (13.42)	26,457 (12.68)	
Histologic Grade			0.05
I, II	2,246 (58.06)	124,439 (59.65)	
III	1,622 (41.93)	84,182 (40.35)	
ER Status			0.33
Positive	2,977 (76.96)	159,151 (76.29)	
Negative	891 (23.04)	49,470 (23.71)	
PR Status			0.11
Positive	2,618 (67.68)	138.626 (66.45)	
Negative	1,250 (32.32)	69,995 (33.55)	
Type of Surgery			**<0.01**
Lumpectomy	1,946 (50.31)	122,191 (58.57)	
Mastectomy	1,893 (48.94)	84,292 (40.40)	
None	18 (0.46)	1,685 (0.81)	
Radiation Therapy			**0.01**
Yes	2,128 (55.02)	120,087 (57.56)	
No	1,740(44.98)	88,534(42.44)	

Abbreviations: AJCC, American Joint Committee on Cancer; LN, lymph node; ER, estrogen receptor; PR, progesterone receptor.

a Bold type indicates statistical significance.

b Not married includes never married, divorced, separated, and widowed.

Using the Chi-square test, we found significant differences in the demographic characteristics between the Caucasian and Chinese patients. The Chinese patients had a younger age at diagnosis, a later year of diagnosis, and a higher family income than the Caucasians. In addition, more Chinese patients remained married and resided in metropolitan areas. With regard to tumor characteristics, the Chinese patients seemed more inclined to receive mastectomy than lumpectomy compared with the Caucasians, and fewer Chinese patients underwent radiation therapy. The other tumor characteristics, including laterality, tumor size, lymph node (LN) status, American Joint Committee on Cancer (AJCC) stage, histologic grade, and estrogen receptor (ER) and progesterone receptor (PR) statuses, were similarly distributed between the two races.

### Comparison of Survival between Chinese and Caucasian Patients

**Figure 1:**
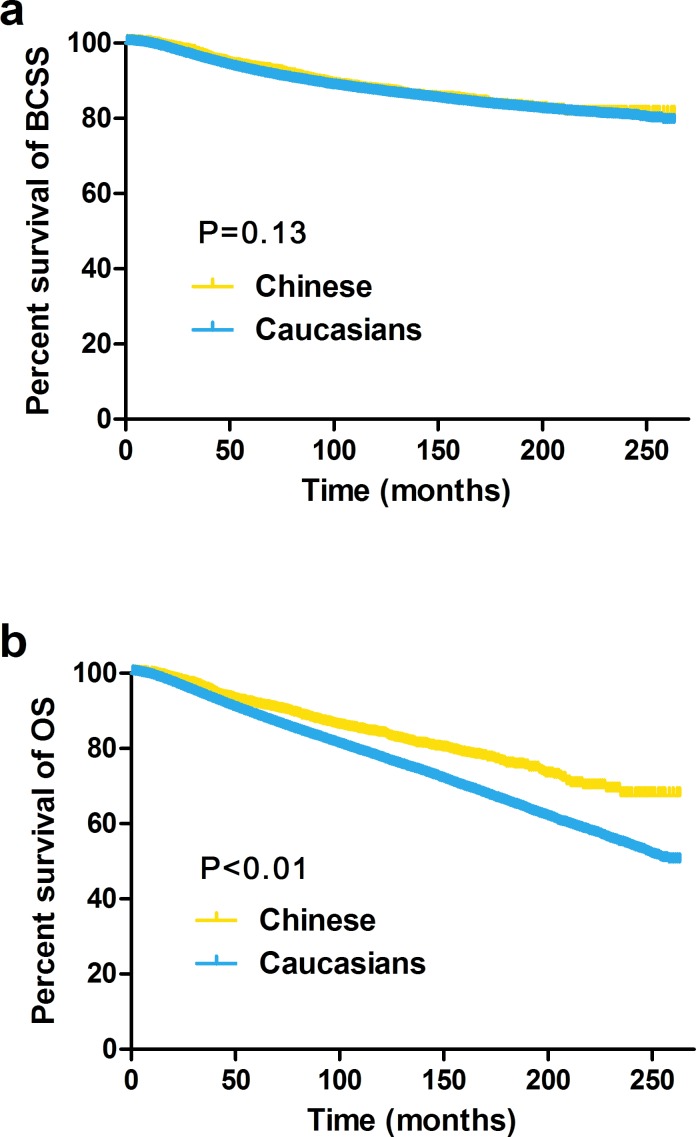
Kaplan–Meier estimates of breast cancer-specific survival (BCSS, a) and overall survival (OS, b) between Caucasian and Chinese female breast cancer patients residing in the US

We first analyzed the unadjusted breast cancer-specific survival (BCSS) and overall survival (OS) rates of the Caucasian and Chinese patients using the Kaplan-Meier method and log-rank test (Figure 1). Compared to the Caucasians, the Chinese patients had significantly better OS rates (P < 0.01) but similar BCSS rates (P=0.13).

The results of the BCSS and OS analyses via univariate and multivariate Cox proportional hazard regression models are shown in Table 2 and Table 3, respectively. The BCSS was not significantly different between the two races (univariate P=0.13, multivariate P=0.10). In contrast, all of the other characteristics, including older age, a later year of diagnosis, continued married status, a higher family income, living in a metropolitan area, a tumor on the right side, a tumor size of 0-2 cm, a negative LN status, higher differentiation, ER and PR positivity, increased acceptance of lumpectomy and receipt of radiation therapy, were independently associated with increased BCSS (Table 2), according to univariate and multivariate analyses. These factors were also related to increased OS (Table 3), and the Chinese patients showed significantly better OS than the Caucasians (using the Caucasians as a reference, univariate: hazard ratio, HR=0.68, 95% confidence interval, CI: 0.63-0.74, P < 0.01; multivariate: HR=0.76, 95% CI: 0.69-0.83, P < 0.01).

**Table 2:** Univariate and multivariate Cox analysis of breast cancer-specific survival

Variables	Univariate		Multivariate^a^	
	HR (95% CI)	P^b^	HR (95% CI)	P^b^
Race		0.13		0.07
Caucasian	1		1	
Chinese	0.92 (0.83-1.02)		0.90 (0.81-1.01)	
Age at Diagnosis (years)		**<0.01**		**<0.01**
20-39	1		1	
40-59	0.59 (0.56-0.61)		0.85 (0.82-0.89)	
60-79	0.59 (0.56-0.61)		1.10 (1.05-1.15)	
Year of Diagnosis		**<0.01**		**<0.01**
1990-1999	1		1	
2000-2009	0.65 (0.63-0.66)		0.66 (0.64-0.68)	
Marital Status		**<0.01**		**<0.01**
Married	1		1	
Not married^c^	1.21 (1.18-1.24)		1.18 (1.15-1.21)	
Family Income ($)		**<0.01**		**<0.01**
<61,790	1		1	
61,790-69,700	0.99 (0.96-1.03)	0.70	0.94 (0.91-0.98)	
69,700-85,140	0.87 (0.83-0.90)		0.91 (0.87-0.95)	
≥85,140	0.81 (0.78-0.85)		0.87 (0.83-0.90)	
County Type		**<0.01**		**0.04**
Metropolitan	1		I	
Nonmetropolitan	1.14 (1.10-1.19)		1.05 (1.00-1.10)	
Laterality		**0.01**		**0.02**
Left	1		1	
Right	0.96 (0.93-0.98)		0.97 (0.94-1.00)	
Tumor Size (cm)		**<0.01**		**<0.01**
0-2	1		1	
2-5	3.61 (3.50-3.72)		1.65 (1.59-1.72)	
>5	8.33 (8.01-8.66)		2.35 (2.21-2.48)	
LN Status		**<0.01**		**<0.01**
Negative	1		1	
1-3 positive	2.37 (2.30-2.45)		1.50 (1.44-1.57)	
3 positive	6.60 (6.40-6.81)		2.27 (2.12-2.42)	
Histologic Grade		**<0.01**		**<0.01**
I,II	1		1	
III	3.19 (3.10-3.28)		1.70 (1.65-1.76)	
ER Status		**<0.01**		**<0.01**
Positive	1		1	
Negative	2.58 (2.51-2.65)		1.40 (1.35-1.45)	
PR Status		**<0.01**		**<0.01**
Positive	1		1	
Negative	2.37 (2.31-2.44)		1.38 (1.33-1.43)	
Type of Surgery		**<0.01**		**<0.01**
Lumpectomy	1		1	
Mastectomy	2.38 (2.32-2.45)		1.12 (1.08-1.16)	
None	6.86 (6.24-7.55)		3.47 (3.143.83)	
Radiation Therapy		**<0.01**		**<0.01**
Yes	1		1	
No	1.37 (1.33-1.40)		1.17 (1.13-1.21)	

Abbreviations: AJCC, American Joint Committee on Cancer; ER, estrogen receptor; LN, lymph node; PR, progesterone receptor.

a Adjusted by Cox proportional hazard regression model including all factors, as categorized in Table 2.

b Bold type indicates statistical significance.

c Not married includes never married, divorced, separated, and widowed.

**Table 3:** Univariate and multivariate Cox analyses of overall survival

Variables	Univariate		Multivariate^a^	
	HR (95% CI)	p^b^	HR (95% CI)	p^b^
Race		**<0.01**		**<0.01**
Caucasian	1		1	
Chinese	0.68 (0.63-0.74)		0.76 (0.69-0.83)	
Age at Diagnosis (y)		**<0.01**		**<0.01**
20-39	1		1	
40-59	0.69 (0.67-0.72)		0.89 (0.86-0.93)	
60-79	1.54 (1.48-1.60)		2.21 (2.12-2.30)	
Year of Diagnosis		**<0.01**		**<0.01**
1990-1999	1		1	
2000-2009	0.69 (0.67-0.70)		0.72 (0.71-0.74)	
Marital Status		**<0.01**		**<0.01**
Married	1		1	
Not married^c^	1.57 (1.54-1.60)		1.40 (1.37-1.43)	
Family Income ($)		**<0.01**		**<0.01**
<61,790	1		1	
61,790-69,700	0.92 (0.90-0.95)		0.90 (0.88-0.93)	
69,700-85,140	0.82 (0.80-0.85)		0.87 (0.85-0.90)	
≥85,140	0.76 (0.74-0.78)		0.83 (0.81-0.86)	
County Type		**<0.01**		0.99
Metropolitan	1		1	
Nonmetropolitan	1.18 (1.15-1.22)		1.00 (0.97-1.03)	
Laterality		**0.01**		0.17
Left	1		1	
Right	0.97 (0.96-0.99)		0.99 (0.97-1.01)	
Tumor Size (cm)		**<0.01**		**<0.01**
0-2	1		1	
2-5	2.06 (2.02-2.11)		1.56 (1.51-1.61)	
>5	4.15 (4.02-4.28)		2.13 (2.13-2.35)	
LN Status		**<0.01**		**<0.01**
Negative	1		1	
1-3 positive	1.45 (1.42-1.48)		1.32 (1.27-1.36)	
3 positive	3.23 (3.15-3.31)		1.89 (1.79-2.00)	
Histologic Grade		**<0.01**		**<0.01**
I,II	1		1	
III	1.75 (1.71-1.78)		1.30 (1.27-1.33)	
ER Status		**<0.01**		**<0.01**
Positive	1		1	
Negative	1.62 (1.59-1.66)		1.23 (1.20-1.27)	
PR Status		**<0.01**		**<0.01**
Positive	1		1	
Negative	1.62 (1.59-1.65)		1.23 (1.20-1.26)	
Type of Surgery		**<0.01**		**<0.01**
Lumpectomy	1		1	
Mastectomy	1.91 (1.87-1.95)		1.09(1.06-1.11)	
None	5.01 (4.63-5.42)		3.02 (2.78-3.28)	
Radiation Therapy		**<0.01**		**<0.01**
Yes	1		1	
No	1.53 (1.50-1.56)		1.23(1.20-1.26)	

Abbreviations: AJCC, American Joint Committee on Cancer, ER, estrogen receptor; LN, lymph node; PR, progesterone receptor.

a Adjusted by Cox proportional hazard regression model including all factors, as categorized in Table 2.

b Bold type indicates statistical significance.

c Not married includes never married, divorced, separated, and widowed.

### Comparison of Survival by Stratification Analysis

We considered that there might be confounding factors affecting breast cancer outcomes between the Caucasian and Chinese patients. Therefore, we further performed multivariate analysis, stratifying according to the potential characteristics (Table 4). Although there was no difference between the two races with regard to BCSS, patients between the ages of 60 and 79 years old (using Caucasian as reference: HR=0.73, 95% CI: 0.59-0.91, P < 0.01), those with a tumor size of 0-2 cm (HR=0.77, 95% CI: 0.63-0.93, P < 0.01), and those who were ER-negative (HR=0.81, 95% CI: 0.69-0.96, P=0.02) and PR-negative (HR=0.83, 95% CI: 0.71-0.96, P=0.01) showed significant differences in BCSS, suggesting that the Chinese patients in these subgroups had better survival than the Caucasians. In contrast, most of the stratified subgroups showed significant differences in OS, except for the patients aged 60-79 years old and those with a tumor size of >5 cm, 3 positive LNs, and no history of surgery (P > 0.05). The Chinese patients in these groups did not exhibit superior OS rates to their Caucasian counterparts.

**Table 4:** Multivariate Cox proportional hazard regression model of breast cancer-specific survival and overall-survival comparing Chinese with Caucasians stratified by clinicopathologic variables

Variables^b^	Caucasian vs. Chinese^a^			
	BCSS		OS	
	HR (95% CI)	P^c^	HR (95% CI)	P^c^
Age at Diagnosis (y)				
20-39	0.83 (0.64-1.08)	0.16	0.81 (0.62-1.04)	0.10
40-59	1.03 (0.89-1.18)	0.72	0.93 (0.82-1.06)	0.26
60-79	0.73 (0.59-0.91)	**<0.01**	0.61 (0.52-0.70)	**<0.01**
Tumor Size (cm)				
0-2	0.77 (0.63-0.93)	**<0.01**	0.64 (0.55-0.74)	**<0.01**
2-5	0.94 (0.81-1.10)	0.44	0.83 (0.72-0.94)	**<0.01**
>5	0.94 (0.73-1.21)	0.63	1.06 (0.83-1.36)	0.63
LN Status				
Negative	0.85 (0.71-1.02)	0.09	0.66 (0.57-0.76)	**<0.01**
1-3 positive	1.00 (0.83-1.21)	0.99	0.83 (0.70-0.98)	**0.03**
3 positive	0.89 (0.74-1.06)	0.19	0.87 (0.73-1.02)	0.09
Histologic Grade				
I,II	0.83 (0.68-1.01)	0.07	0.64 (0.55-0.73)	**<0.01**
III	0.93 (0.82-1.06)	0.28	0.85 (0.76-0.95)	**0.01**
ER Status				
Positive	0.98 (0.85-1.12)	0.71	0.77 (0.68-0.86)	**<0.01**
Negative	0.81 (0.69-0.96)	**0.02**	0.76 (0.65-0.89)	**<0.01**
PR Status				
Positive	0.99 (0.85-1.14)	0.84	0.76 (0.67-0.86)	**<0.01**
Negative	0.83 (0.71-0.96)	**0.01**	0.76 (0.66-0.86)	**<0.01**
Laterality				
Left	0.91 (0.78-1.05)	0.20	0.76 (0.67-0.87)	**<0.01**
Right	0.90 (0.77-1.05)	0.18	0.75 (0.66-0.86)	**<0.01**
Type of Surgery				
Lumpectomy	0.98 (0.81-1.18)	0.79	0.77 (0.66-0.91)	**<0.01**
Mastectomy	0.88 (0.77-1.00)	0.06	0.75 (0.67-0.84)	**<0.01**
None	1.28 (0.57-2.88)	0.56	1.22 (0.58-2.59)	0.60
Radiation Therapy				
Yes	0.95 (0.82-1.10)	0.49	0.82 (0.73-0.94)	**<0.01**
No	0.86 (0.73-1.00)	0.06	0.70 (0.61-0.80)	**<0.01**

Abbreviations: ER, estrogen receptor; LN, lymph node; PR, progesterone receptor.

a Using Caucasian as a reference.

b Adjusted by Cox proportional hazard regression model including all factors, as categorized in Table 2.

c Bold type indicates statistical significance.

## DISCUSSION

In our evaluation of more than 200000 primary breast cancer patients reported to population-based cancer registries across the US by the SEER program, we found that the BCSS of Chinese patients living in the US was similar to that of Caucasians. Previous studies that have mainly focused on all Asians living in the US, investigating disparities among unique Asian subgroups, have reported similar results to those of the present study [4, 7], and our findings contributed to these previous studies by providing a comprehensive assessment of differences in breast cancer outcomes between Caucasian and Chinese women residing in the US. Moreover, we found that the Chinese patients had distinctively better OS than the Caucasians, even when stratified by traditional clinicopathologic characteristics, including age, year of diagnosis, marital status, family income, type of residential county, tumor size, LN status, ER and PR statuses, histological grade, surgical treatment and radiation therapy.

Chinese immigrants are considered to have worse socioeconomic status (SES) than their Caucasian counterparts [8], suggesting that they might have limited access to medical insurance and appropriate treatments. In addition, they appear less likely to have their cancer-related issues resolved due to their traditional cultural beliefs [9, 10]. Interestingly, the Chinese patients in our study had much higher family incomes than the Caucasians, and more of them resided in metropolitan counties. Furthermore, they received breast-conserving surgery (BCS) (50.31%, Table 2) much more frequently than the Chinese natives did (12.2%, [11]). Additionally, the rates of BCS and radiation therapy for the Chinese patients residing in the US were similar to those of their Caucasian counterparts (BCS: 50.31 vs. 58.57; radiation therapy: 55.02 vs. 57.56), although the differences were significant. Considering these findings, we inferred that Chinese patients living in the US, or at least those who were registered in the SEER database, represented a well-educated, higher SES group of Chinese individuals who were able to obtain optimal medical resources similar to those obtained by Caucasians. Despite discrepancies in the demographic characteristics, there were no significant differences in tumor characteristics, including tumor size, LN status, ER and PR statuses, histological grade, and AJCC stage. Considering the differences in tumor biology between these two ethnicities, we suggest that the worse survival rate of Chinese patients living in China can mainly be attributed to worse SES and not tumor behavior itself. Chinese patients may experience improved outcomes with more advanced treatment. This notion is supported by the prediction that the future survival curve in China will be similar to that in Western countries with the development of the Chinese economy [1].

In our investigation, we found that the Chinese patients had a higher OS rate than the Caucasians, consistent with previous findings obtained from a study of Asian women aged 18-39 years old, which showed that Chinese Americans have better 5-year and 10-year OS rates than Caucasians do [12]. After stratifying by potentially confounding factors, this advantage still existed, except for those patients aged 60-79 old and those with a tumor size of >5 cm, 3 positive LNs, and no history of surgery. The superior OS of the Chinese patients was markedly attenuated in these groups, which might have been due to their more advanced and aggressive breast cancers, resulting in poor prognoses that neutralized the increase in OS.

Because the BCSS rates were similar between the two races assessed in our study, the difference in OS was not caused by breast cancer-related deaths. Notably, the overall age distribution of the Chinese women was less than that of the Caucasians (Table 2), which might have been partially due to immigration patterns because the Chinese population experienced a rapid increase in younger-aged individuals in the latter half of the twentieth century [12]. Additionally, this finding reflects that Chinese women are typically younger at the time of breast cancer diagnosis [11]. Due to their younger ages, Chinese women might have fewer complications, such as heart disease, cerebrovascular disease, and chronic lower respiratory disease, resulting in greater OS. However, in our further stratification analysis, the patients aged 20-39 and 40-59 years old did not exhibit any differences in survival, whereas the Chinese patients aged 60-79 years old had better survival than the Caucasians did, in terms of both BCSS and OS. A possible explanation for this finding was that older Chinese patients might be more inclined to adopt a traditional Chinese lifestyle, resulting in reduced fat intake and increased physical activity. With healthy life habits, older Chinese patients achieve a lower body mass index and a reduction in obesity, both of which are associated with improved breast cancer survival [13, 14].

Our study had the advantage of including a sizable number of breast cancer patients reported to cancer registries, with near completeness in terms of registration and follow-up data [15]. These large numbers allowed us to examine racial differences in survival among subgroups of patients with different demographic and tumor characteristics. There are clearly important limitations inherent in using a large population-based dataset. Data pertaining to SES, family history of breast cancer, lifestyle factors, human epidermal growth factor receptor 2 status, medical insurance, and the administration of neoadjuvant or adjuvant systemic therapy were limited, preventing us from evaluating these factors as potential confounders or effect modifiers of the relationships observed. The long duration of our study (1990-2009) might have affected the results due to missing data or changes in management. However, neither of these factors would be expected to result in a sufficiently strong bias to override the known impacts of tumor size, LN status, age at diagnosis, race, and SES, all of which were included in the multivariate model.

In conclusion, our study demonstrated that the presenting demographic features of breast cancer patients differed between Caucasian and Chinese women residing in the US. However, the tumor characteristics in the two groups were similar, suggesting that Chinese patients will have similar outcomes once they attain better medical resources. Regardless of these factors, the Chinese women had better OS than their Caucasian counterparts. Further pre-clinical and clinical studies should be conducted to confirm these conclusions and to clarify the underlying mechanisms.

## MATERIALS AND METHODS

### Ethical statement

This study was approved by the Ethical Committee of the Shanghai Cancer Center of Fudan University. The data released from the SEER database did not require informed patient consent because cancer is a reportable disease in every state in the US.

### Patient selection

The data for this study were derived from the SEER program of the US National Cancer Institute (SEER 18 Regs Research Data and Hurricane Katrina Impacted Louisiana Cases, Nov 2013 Sub [1973-2011 varying]). We selected female patients diagnosed with primary invasive breast cancer from January 1, 1990, through December 31, 2009. Race/ethnicity was our primary exposure of interest, and we selected Caucasian and Chinese individuals residing in the US according to the SEER algorithm [16]. Women with other combinations of race/ethnicity were excluded.

The specific inclusion criteria were as follows: female sex, Chinese or Caucasian race/ethnicity, age of diagnosis between 20 and 79 years old, diagnosis between 1990 and 2009, breast cancer as the primary and only cancer diagnosis, unilateral breast cancer, pathologically confirmed infiltrating ductal carcinoma (IDC, ICD-O-3 8500/3), AJCC stages I to III, histological grades I to III, and known tumor size, as well as LN status, ER and PR statuses. Women who were diagnosed with breast cancer at death or by autopsy only and those with other first primary cancers, *in situ* disease, histological grade IV (SEER program code: undifferentiated or anaplastic), and no record of surgery type or radiation therapy were excluded from this analysis. Patients diagnosed with breast cancer before 1990 were not included because the SEER database did not record data on ER and PR statuses until 1990. Additionally, patients diagnosed with breast cancer after 2010 were not included because the database was only updated through December 31, 2010, and we wanted to ensure an adequate follow-up duration.

### Statistical analysis

The covariates included in our analyses were limited to those available in the SEER program data. Demographic statistics included race, age at diagnosis, year of diagnosis, marital status, family income, and county metropolitan status. Tumor characteristics included laterality, tumor size, lymph node status, AJCC stage, histological grade, ER status, PR status, surgery type and radiotherapy. The primary outcomes of our study were BCSS and OS. BCSS was defined as the time from the date of diagnosis to the date of death due to breast cancer or the last follow-up, and OS was measured from the date of diagnosis to the date of death due to all causes (including breast cancer) or the last follow-up.

Patient and tumor characteristics between the different races were compared using Pearson's Chi-square test. Survival curves were generated using the Kaplan-Meier method, and the log-rank test was performed to compare the unadjusted BCSS and OS rates between the two races. Adjusted HRs with 95% CIs were estimated using Cox proportional hazard regression models. P-values of <0.05 were considered significant. SPSS software, version 18.0 (SPSS, Inc., Chicago, IL, US), was used for all of the analyses.
